# The Role of Convex Probe Endobronchial Ultrasound Guided Transbronchial Needle Aspiration in the Diagnosis of Malignant Mediastinal and Hilar Lymph Nodes

**DOI:** 10.5812/iranjradiol.3882

**Published:** 2012-11-20

**Authors:** Benan Caglayan, Banu Salepci, Ilgaz Dogusoy, Ali Fidan, Sevda Sener Comert, Nesrin Kiral, Dilek Yavuzer, Gulsen Sarac

**Affiliations:** 1 Department of Pulmonary Diseases, Dr. Lütfi Kırdar Kartal Training and Research Hospital, Istanbul, Turkey; 2 Department of Thoracic Surgery, Dr. Siyami Ersek Thoracic and Cardiovascular Surgery Training and Research Hospital, Istanbul, Turkey; 3 Department of Pathology, Dr. Lütfi Kırdar Kartal Training and Research Hospital, Istanbul, Turkey

**Keywords:** Ultrasonography, Lung Cancer, Mediastinal Neoplasms, Lymph Nodes

## Abstract

**Background:**

In the diagnosis of malignant lymph nodes (LNs) and staging of lung cancer, sampling of mediastinal and hilar LNs is essential. Mediastinoscopy is known as the gold standard. Convex probe (CP) endobronchial ultrasound (EBUS)-guided transbronchial needle aspiration (TBNA) is a noninvasive and highly sensitive diagnostic method in mediastinal and hilar LN sampling.

**Objectives:**

Evaluating the role of CP-EBUS-guided TBNA in the diagnosis of mediastinal and hilar LNs suspicious of malignancy.

**Patients and Methods:**

One hundred twenty patients with a known lung malignancy or hilar/mediastinal LNs detected by thoracic computed tomography (CT) and/or positron emission tomography (PET)-CT suspicious for malignancy were included in this prospective study. The procedure was performed by Olympus 7.5 MHz CP endoscope and EU C2000 processor by the oral route under topical anesthesia and conscious sedation. After visualization of LNs, their dimensions were recorded. Aspiration was considered as “insufficient” if there were inadequate lymphocytes on the smears. Diagnosis of “malignancy” on cytologic examination was considered as the “final diagnosis”. If diagnosis was negative for malignancy, more invasive procedures were performed to confirm the diagnosis.

**Results:**

Twenty four females and 96 male patients (mean age, 57.8 ± 9.1) were included. A total of 177 LN stations were aspirated in 120 patients. In 82 patients, the diagnosis was malignant by EBUS-guided TBNA and in the remaining 38; the diagnosis was established by further invasive procedures. Of the 38 EBUS-guided TBNA negative patients, 28 were diagnosed as non-malignant and 10 were malignant. The sensitivity, diagnostic accuracy and negative predictive value of CP EBUS-guided TBNA were 89.1%, 91.6% and 73.6%, respectively. No major complications were seen.

**Conclusion:**

As an alternative method to mediastinoscopy, EBUS-guided TBNA is a safe and noninvasive procedure with high sensitivity in the diagnosis of malignant mediastinal LNs.

## 1. Background

Mediastinal and hilar LN sampling is essential in the diagnosis of malignant mediastinal or hilar lymph nodes and the staging of lung cancer. Lung cancer remains the first cause of cancer-related death with an overall 5-year survival rate of 15%. The best hope for cure is for patients with potentially resectable disease, but even in these cases, a 5-year survival rate of only 40-50% has been mentioned (1). The most common type of lung cancer is non-small cell lung cancer, the prognosis and treatment of which is basically determined by disease stage (2). The American College of Chest Physicians (ACCP) and the European Society of Thoracic Surgeons (ESTS) recommend establishing staging by means of tissue diagnosis from the suspicious lymph nodes (3, 4). On the other hand, in the presence of a known extrapulmonary tumor, metastasis to the mediastinal lymph nodes changes the stage, prognosis and the treatment strategy of the disease. Metastasis to mediastinal lymph nodes may also indicate recurrence in a patient whose disease is under control. Although mediastinoscopy and thoracoscopy are considered as the reference techniques for the tissue diagnosis of mediastinal or hilar LNs, bronchoscopic minimal invasive methods are also used for the same purposes. Thoracic CT is recommended by the American Thoracic Society (ATS) as one of the non-invasive staging methods in the assessment of the mediastinal involvement. Although LNs with a short axis diameter of more than 1 cm on thoracic CT scan are suspicious of malignancy, sensitivity and specificity are considerably low. 18F-fluoro-2-deoxy-D-glucose positron emission tomography (FDG-PET), a metabolic imaging method, is commonly used for the staging of lung cancer. However, owing to the low specificity of this method, cytopathologic examination is recommended in positive cases (5-8).

**Figure 1:**
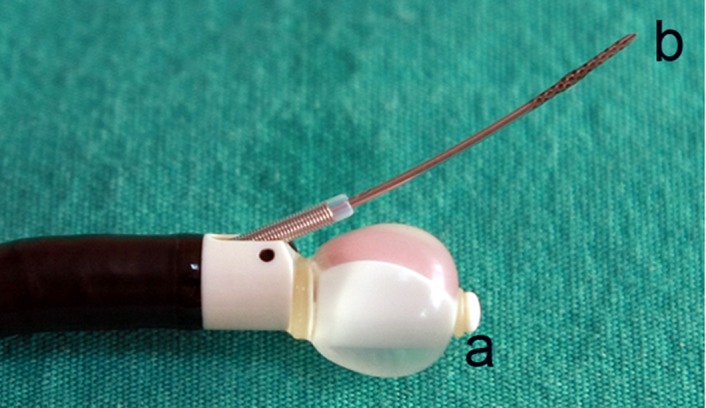
Distal tip of dedicated bronchoscope with the curved array transducer covered with a saline inflated baloon (a) and echogenic needle pushed out of the bronchoscope (b)

The diagnostic value of conventional TBNA in the tissue diagnosis of mediastinal and hilar LNs is between 40% and 50%. Furthermore, this ratio changes between 15% and 85% depending on the size and location of the LN. Thus, the use of this method is decreased particularly in small LNs (9, 10). Mediastinoscopy which is used for lung cancer staging still maintains its importance as a reference technique. The disadvantages of mediastinoscopy which has a quite high sensitivity (90% to 95%) are that it is an invasive method requiring general anesthesia and it cannot reach all mediastinal LNs; therefore, additional staging methods such as thoracoscopy are needed in many cases. Furthermore, the fact that mediastinoscopy has a 2-3% complication rate may be considered as a disadvantage (6, 7, 11). Ultrasound has been increasingly incorporated into diagnostic and therapeutic modalities. Ultrasound technology may be employed via a probe inserted through the working channel (radial probe EBUS) or incorporated into the distal end of the bronchoscope (convex probe EBUS), the latter allowing real-time biopsy. The convex probe EBUS bronchoscope, which incorporates the ultrasound transducer at its distal end, utilizes a fixed array of transducers aligned in a curvilinear pattern. This generates a 50° image parallel to the long axis of the bronchoscope. Use of a 7.5-MHz frequency allows deeper tissue penetration. Using the water-filled balloon can improve the image quality (Figure 1). Power Doppler capability differentiates the tissue from the vascular structure. Ultrasound and the white-light bronchoscopic images can be viewed simultaneously (12).

Recently introduced transbronchial and transesophageal needle aspirations performed under the guidance of real-time endoscopic ultrasonography, which are minimally invasive methods and do not require general anesthesia, can sample many LNs which cannot be reached by mediastinoscopy. This has been a significant breakthrough in the staging of lung cancer. The sensitivity of EBUS-guided TBNA for the detection of malignant LNs and the diagnostic value were found to be higher than 90% (5, 7, 13, 14).

## 2. Objectives

The aim of the present study is to evaluate the role of CP-EBUS guided TBNA in the diagnosis of the nature of mediastinal and hilar lymph nodes in patients with a known lung malignancy or hilar/mediastinal LN detected by thoracic CT and/or PET-CT suspicious for malignancy.

## 3. Patients and Methods

### 3.1. Study Design

The patients with a known lung malignancy or hilar/mediastinal lymph nodes detected by thoracic CT and/or PET-CT suspicious for malignancy that were followed by our department from October 2008 to November 2010 were included in this prospective study. All patients underwent CP-EBUS-guided TBNA for staging or diagnostic purposes. Mediastinoscopy or other invasive procedures were performed if CP-EBUS-guided TBNA was negative for malignancy or did not provide representative material. The study protocol was approved by the local ethics committee and written informed consent was obtained from all patients included in the study.

### 3.2. Patients

The patients were eligible for this study if they were older than 16 years of age in the presence of a known lung malignancy with hilar/mediastinal lymph nodes with a short axis more than 1 cm on thoracic CT scan and/or PET-CT suspicious for malignancy or hilar and/or mediastinal lymph nodes positive on PET/CT scan without regarding the diameter suspicious for malignancy. If informed consent was not obtained or an uncontrolled coagulopathy was present (platelets < 20000/mm3, INR > 1.3), the patient was excluded.

### 3.3. Convex Probe EBUS- Guided TBNA

**Figure 2:**
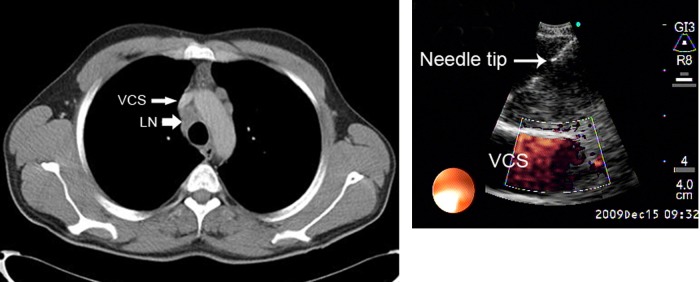
A, Right lower paratracheal lymph node. The tip of the needle is shown with an arrow. The echogenic view under the tip of the needle is an internal echo of the lymph node; B, The correlative CT image of the right lower paratracheal lymph node. Abbreviations: LN; lymph node, VCS; vena cava superior

CP-EBUS-guided TBNA from hilar and mediastinal lymph nodes were performed after physical examination, chest X-ray and thoracic CT. Routine biochemical analysis, pulmonary function tests and PET-CT were done if indicated. EBUS-guided TBNA examinations were performed in all cases at the pulmonary department as an outpatient procedure in a dedicated bronchoscopy suit with a 7.5 MHz, BF-UC160F (Olympus Optical Co., Tokyo, Japan, approved by FDA) convex probe bronchoscope and EU C2000 processor (Olympus, Tokyo, Japan) by oral route and in the supine position under local anesthesia with lidocaine and conscious sedation with intravenous midazolam. Lymph nodes were identified according to the Mountain’s regional lymph node classification system (15). The lymph node stations of 2, 4, 7, 10 and 11 were evaluated systematically. The dimensions of the lymph nodes seen on the CP-EBUS were recorded from frozen ultrasound images. In the presence of any lymph node with a short axis greater than 0.5 cm, even if CT- and PET-CT were negative, EBUS-guided TBNA was performed with real-time imaging (Figure 2). Olympus 22 -gauge NA-201SX-4022-C needle (approved by FDA) was used for the procedure. During the process for every detected lymph node; short and long axis diameters, station of the lymph node, number of passes per patient and per lymph node were recorded for each patient. To avoid contamination in lung cancer patients, N3 nodes were sampled first and then N2 nodes were punctured. 

We could not perform pathologic rapid on-site evaluation (ROSE). The materials obtained by EBUS-guided TBNA were fixed in 95% alcohol and sent to the pathology laboratory after smearing on slides. Aspiration specimen was considered “insufficient” if there were not an adequate number of lymphocytes on the smear. Diagnosis as “malignant” in cytologic examination was considered as the “final diagnosis”. For the patients whose EBUS-guided TBNA results were negative for malignancy, more invasive procedures such as mediastinoscopy were done to confirm the diagnosis. 

### 3.4. Statistics

The sensitivity, diagnostic value and negative predictive value of the EBUS-guided TBNA in the diagnosis of malignant mediastinal and hilar lymph nodes were calculated using the standard definitions. Fisher’s exact test and chi-square test were used for the statistical analysis.

## 4. Results

Of the 135 patients suspected of malignancy and having hilar and/or mediastinal lymphadenopthy in CT and/or PET-CT, 120 patients meeting the inclusion criteria were evaluated from October 2008 to November 2010. Of these 120 patients, 96 (80%) were males and 24 (20%) were females. The mean age was 57.8 ± 9.1 years (34-81). In 99 of the cases, the diagnosis of lung cancer was established or lung cancer was radiologically suspected. In the remaining 21 cases, diagnosis of other system cancers most commonly breast cancer was confirmed (Table 1). CP-EBUS-guided TBNA was performed for diagnostic, staging, both diagnostic and staging, or restaging purposes in 55, 29, 34 and 2 cases, respectively. In all cases, aspiration was performed from 177 lymph node stations (38 hilar and 139 mediastinal). The average short axis diameter of sampled LNs was 1.63 cm (min, 0.3; max, 4.0 cm). Totally, 322 aspirations were performed from 177 LN stations. The mean number of LN station aspirations per patients was 1.47. The mean number of aspirations per patient was 2.68 and the mean number of aspiration per LN was 1.81.

**Table 1:** Distribution of Patients Included in the Study in Terms of Primary Cancer

	Number of Cases, No (%)
** Lung**	99 (82.5)
** Breast**	10 (8.3)
** Colorectal**	3 (2.5)
** Uterus/Ovary**	3 (2.5)
** RCC**	2 (1.6)
** Others**	3 (2.5)

Abbreviation: RCC; renal cell carcinoma

**Figure 3:**
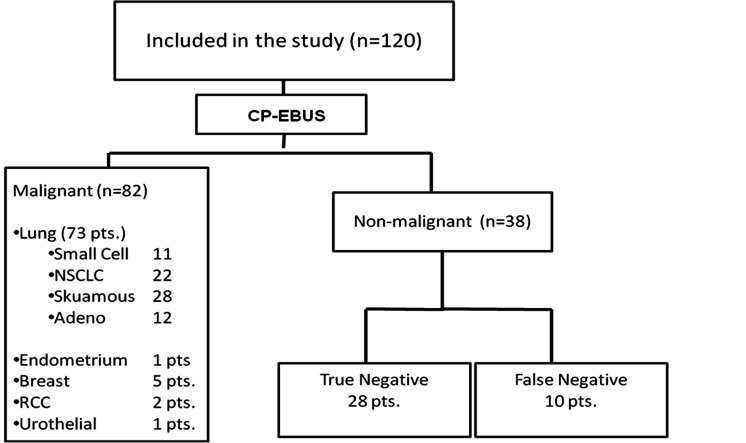
Flow diagram of patients enrolled in the study

Among 120 cases, the EBUS-guided TBNA specimens of five cases did not contain a sufficient number of lymphocytes and thus were considered as insufficient. Malignant cells were seen in 82 of the remaining 115 cases and these were considered as true positive. Unnecessary mediastinoscopy was prevented in these cases by EBUS-TBNA. Although there was sufficient material in 33 cases, no malignant cells were observed. In these 38 cases, more invasive procedures were performed for definitive diagnosis or the patients were followed up for the mediastinal LNs for at least six months. Definite diagnosis was determined as malignant in 10 of 38 cases with negative EBUS-guided TBNA and the final diagnosis was established by invasive procedures in nine of theses 10 cases. In one case, LN disappeared with chemotherapy during follow-up. Of the 28 cases (true negatives), in which no malignancy was detected by means of invasive interventions, diagnoses of reactive adenitis, tuberculosis and sarcoidosis were established in 25, two and one case, respectively (Figure 3), (Table 2). Based on the above-mentioned results in 120 cases, the sensitivity, diagnostic accuracy and negative predictive value (NPV) of CP-EBUS-guided TBNA in the diagnosis of malignant hilar or mediastinal LNs were 89.1%, 91.6% and 73.6%, respectively. When assessed according to LN stations, the stations in which aspiration was most commonly performed in our study were 7, 4R and 11L, respectively. The relation between the short axis diameter of the LN and the sensitivity of EBUS-guided TBNA was investigated; 64 LNs had less than 1 cm short axis diameter and 100 LNs had more than 1 cm short axis diameter and the sensitivities were 86% and 95%, respectively. The relation between the short axis diameter of the LN and the sensitivity of EBUS-guided TBNA was significant (P = 0.043). While the sensitivity of EBUS-guided TBNA was 85.4% in 62 cases with aspirations performed once and twice, it was 98.2% in 58 with aspirations performed thrice or more. The relation between the sensitivity and number of aspirations was significant (P = 0.017). In 65 of the 99 lung cancer cases, EBUS was performed for staging purposes. Ten cases were down-staged and one case was up-staged by EBUS-guided TBNA when compared with PET and PET-CT findings (Table 3). No major complication was observed in our study. The only complication was hemorrhage, which was detected in five patients.

**Table 2:** Distribution of Methods in Which 38 Non-Malignant Patients Were Definitively Diagnosed

	Number of cases	Benign LN	Malignant LN
** Mediastinoscopy**	23	16 RA, 1 Tb	6
** Mediastinoscopy + Thoracotomy**	4	4 RA	-
** Thoracotomy**	6	2 RA, 1 Tb	3
** Follow up**	5	3 RA, 1 Sarc	1
** Total**	38	28 (25 RA, 2 Tb, 1 Sarc.)	10

Abbreviations: LN; lymph node, RA; Reactive adenitis, Sarc; Sarcoidosis, Tb; Tuberculosis

**Table 3:** The Effect of CP-EBUS-Guided TBNA Results on Staging Established by CT/PET

No.	Nodal Status According to PET	Nodal Status According to CP EBUS-TBNA	Change of Stage
** 2**	N3	N0	Down-stage
** 2**	N3	N2	Down-stage
** 4**	N2	N0	Down-stage
** 2**	N1	N0	Down-stage
** 1**	N0	N2	Up-stage

## 5. Discussion

In the presence of a known cancer or cancer suspicion, the cytological and histological diagnosis of the suspicious mediastinal or hilar LNs has a strategic importance in the evaluation of the disease, treatment decision and determination of the prognosis. In non-small cell lung cancer (NSCLC) cases, mediastinal LN is the region in which metastasis is seen most commonly. In the presence of metastatic LN, the recommended therapy is chemoradio therapy and the patient loses the chance of surgery. ACCP recommends performing invasive staging when a lesion with central localization or an abnormal LN in CT/PET is seen (8). Although mediastinoscopy and thoracoscopy are standard methods for the diagnosis of hilar and mediastinal LNs, they have some disadvantages; they require general anesthesia and hospitalization, they have a 0.08% mortality risk and some LN stations cannot be reached by these methods (13). On the other hand, although mediastinoscopy is a method accepted as the gold standard in the staging of lung cancer, in 2005, Little et al. have shown that mediastinoscopy has been performed only in 27% of the 11668 surgically treated NSCLC cases in 729 hospitals and that lymph node biopsy was done in 1054 cases comprising 46.6% of these cases (16). Similarly, Smulders et al. have revealed that mediastinoscopy was performed according to gold standard techniques in 40% and the specimens could be collected from LNs in only 50% of the cases (5, 17).

In contrast to mediastinoscopy, real time CP-EBUS TBNA has many advantages; it is a method that may be performed with sedation in outpatient settings, it has a high sensitivity with a very low morbidity risk and in addition to LN stations 2, 4 and 7a it may help to reach stations 7p, 10 and 11 which are inaccessible by mediastinoscopy (17, 18). In many studies conducted after availability of CP-EBUS in routine practice, the sensitivity of this method in detecting malignant LNs was greater than 90%. In the prospective study of Herth et al. including 502 patients, the sensitivity and NPV of CP-EBUS-guided TBNA in diagnosing malignant mediastinal and hilar LNs were 94 % and 11%, respectively (19). In a study where the efficacy of CT, PET and CP-EBUS in determining the malignant mediastinal lymph nodes in 102 lung cancer cases who were candidates for surgical intervention were evaluated, Yasufuku et al. reported that the sensitivity of the three methods were 76.9%, 80% and 92.3%, respectively and that the specificity of these methods were 55.3%, 70.1% and 100%, respectively. In this study, the diagnostic accuracy for CP-EBUS was reported as 98% (7). In the algorithm Yasufuku et al. developed according to the results of their study, in potentially resectable pulmonary cancer cases in which hilar and mediastinal LNs suspected to be malignant by CT or PET, they recommended that EBUS-guided TBNA should be primarily performed and in case of a negative result, mediastinoscopy should be performed in order to confirm the results. In another study, the same researchers have reported that the sensitivity and the diagnostic value for CP-EBUS-guided TBNA were 95.7% and 97.1%, respectively (20). In our study, the sensitivity, the diagnostic accuracy and the NPV of the CP-EBUS-guided TBNA in the diagnosis of malignant mediastinal and hilar LNs were 89.1%, 91.6% and 73.6%, respectively. Although the results that we have obtained are slightly lower than those reported in the literature, they are satisfactory. We suggest that the reason why our sensitivity and diagnostic value are lower than those in the literature at least could be due to the fact that we could not perform rapid onsite evaluation (ROSE). In recent years, pertinent studies have shown that performing EBUS and EUS TBNA with a single endoscope in the same session can obtain specimens from stations 5, 8, 9 in addition to stations 2, 4, 7, 10, 11. When these two endoscopic methods are performed together, a wider range of sampling may be performed in comparison to mediastinoscopy and hence, more accurate staging can be achieved. In a study by Herth et al. conducted in 2010, evaluating a total of 619 LNs in 139 NSCLC patients, they sampled 390 LNs by EBUS, 299 LNs by EUS and reported a mean LN diameter of 17 mm. In this study, the sensitivity was computed as 92%, 89% and 96%, respectively for EBUS, EUS and EBUS + EUS TBNA (13). Beside this study, in their prospective study containing 150 lung cancer cases, Wallace et al. revealed that the sensitivity (93%) and NPV (97%) was higher when EUS and EBUS-guided TBNA were used in combination compared to their use separately (21). Vilmann et al. reported that the diagnostic value is 100% by combined use of the two methods (22). In the prospective study of Hwangbo, mediastinal metastasis was detected in 38 of 143 patients with EBUS-guided TBNA and mediastinal metastasis was revealed in three additional patients when EUS was performed by means of ultrasonographic bronchoscopy after which the sensitivity increased from 84% to 91% (23).

The diagnosis of the LNs smaller than 1 cm may be difficult by imaging methods such as CT and PET and also EBUS. Furthermore, since it is a blind method, the sensitivity of conventional TBNA in these small LNs is lower. Since these small LNs contain less malignant cells, there may be difficulties also in cytological diagnosis by EBUS-guided TBNA (18). Despite this fact, EBUS-guided TBNA was found considerably successful to demonstrate malignancy in these LNs in the performed studies. In their 100-case series with smaller than 1cm LNs, Herth et al. reported that the mean short axis of LNs was 8.1 mm and that the sensitivity of the method in detecting malignancy was 92.3% (18). In another study conducted by Herth et al. with CP EBUS in cases in which radiologic and PET imaging were found as normal, nine malignant LNs were detected in 97 patients in which sampling was done by EBUS-guided TBNA. A false negative result was obtained in only one case. According to these findings, the sensitivity of EBUS-TBNA in CT- and PET-negative lung cancer cases was 98.9%. In this study, the mean LN short axis diameter has been reported as 7.9 mm (24). When the researchers have interpreted the results of two studies, they have concluded that the clinical staging done only by radiologic imaging and even by metabolic imaging is not sufficient and that EBUS-guided TBNA is necessary for the cases in which surgery is suggested (25). In their study, Vincent et al. performed aspiration for 167 LNs in 113 cases, 71 of whom were primary lung cancer patients. They have obtained sufficient material in 73.7% of them. In this study, thirty five lymph nodes were subcentrimetric and lymphoid material was obtained from 86% of them (5). In our study, for the diagnosis of the LNs smaller than 1 cm and for those larger than 1 cm, the sensitivity of EBUS-guided TBNA was 86% and 95%, respectively reaching a statistically significant difference (P = 0.043). Nevertheless, the sensitivity we have obtained for LNs smaller than 1 cm has been found considerably satisfactory. The sensitivity and specificity of PET and PET-CT which are the most important non-invasive metabolic imaging methods used in mediastinal staging in lung cancer are particularly low in the presence of small LNs. In studies comparing CP-EBUS-guided TBNA with PET, it has been revealed that the stage of the disease has changed by performing EBUS-guided TBNA after PET (5, 7, 25). In our study, among 95 cases in which PET was performed, the sensitivity and specificity of PET were found as 94.2% and 15.3%, and the sensitivity and the specificity of CP-EBUS-guided TBNA were found as 88.5% and 100%, respectively. In our study, in contrast to the study of Yasufuku (7), the reason why PET sensitivity was higher than EBUS-guided TBNA originates from the design of the study. Since we had included cases with CT- and/or PET-positive mediastinal and/or hilar LNs, the majority of 95 cases for which PET was performed in our series were PET-positive and PET was negative only in seven cases. For the same reason, the specificity of PET was also found to be low in our series. In their retrospective study, Vincent et al. revealed that 15.9% of the cases were down-staged and 9.7% of the cases were up-staged by EBUS in CT- and PET-positive cases. In four cases which were up-staged, the EBUS-guided TBNA results of the contralateral mediastinal LNs was positive which were CT-/PET-negative. In the remaining seven cases, the primary tumor was in the extrapulmonary systems. In this study, the EBUS sensitivity has been reported as 98.7% (5). In our study, PET and CP-EBUS were performed for the purpose of NSCLC staging in 65 cases and the stage of the disease has been changed in 11 of 65 cases by EBUS-guided TBNA. In our series, the stage of the disease changed in 10 (15.3%) cases leading to down-staging and in one (1.5%) case leading to up-staging. Until now, no major complication has been reported in the published studies concerning CP-EBUS-guided TBNA. We also did not detect any complication in our series except minimal hemorrhage. Regarding literature data and the results that we obtained from our study, it may be stated that CP-EBUS-guided TBNA is an effective and reliable method in the diagnosis of malignant mediastinal and hilar LNs and in the staging of lung cancer. In addition to these findings, when the results concerning mediastinoscopy in the study of Little and Smulders mentioned at the beginning of the discussion are considered, it may be suggested that EBUS-guided TBNA is a true alternative for mediastinoscopy in the diagnosis of malignant mediastinal and hilar LNs and in nodal staging of lung cancer and that this minimal invasive method should be performed particularly in all potentially operable cases.
